# Supplementary material for: Exploring the Regulation of Cytochrome P450 in SH-SY5Y Cells: Implications for the Onset of Neurodegenerative Diseases
Source: Int J Mol Sci. 2024 Jul 6;25(13):7439. doi: 10.3390/ijms25137439 (PMC11242626; doi:10.3390/ijms25137439)
Supplement: Supplementary file 1 [file ijms-25-07439-s001.zip › ijms-2950327-supplementary.pdf]

Supplementary Materials

Exploring the regulation of cytochrome P450 in SH-SY5Y cells: implications for the onset of neurodegenerative diseases

Alice Pifferi[1], Elda Chiaino[1], Jesus Fernandez-Abascal[2, 3], Aoife C. Bannon[1], Gavin P. Davey[4], Maria Frosini[1*], Massimo Valoti[1]

1 Dipartimento di Scienze della Vita, Università di Siena, viale A. Moro 2, 53100 Siena, Italy;
2 Andalusian Centre for Developmental Biology (CABD), CSIC-Universidad Pablo de Olavide-Junta de Andalucía, Carretera de Utrera
km 1, 41013 Sevilla, Spain (present address);
3. [b]Department of Molecular Biology and Biochemical Engineering, Universidad Pablo de Olavide, Carretera de Utrera km 1, 41013 Seville, Spain (present address);
4. Department of Neurodegeneration and Systems Biology, School of Biochemistry and Immunology, Trinity College Dublin, 3533645 Dublin, Ireland;
* Correspondence: maria.frosini@unisi.it

[*]**Corresponding author**:
Prof. Maria Frosini,
University of Siena, Dipartimento di Scienze della Vita,
viale A. Moro 2, lotto C,
53100 Siena, Italy.
Phone: +390577235354;
E-mail address: maria.frosini@unisi.it

Table S1. Antibodies used for Western Blot experiments

Target	Source	*Cat. Numb.*	*Lot*	Host	Concentration
NeuN	Invitrogen	PA5-37407	Not available	Rabbit	1:1000
Synaptophysin	Sigma Merck	SAB4502906	Not available	Rabbit	1:1500
β tubulin III	Sigma Merc	SAB700544	Not available	Mouse	1:1000
MAO-A	Sigma Merck	SAB1410774	15211	Rabbit	1:100
MAO-B	Santa Cruz Biotechnology	SC515354	D2321	Mouse	1:100
DAT	Sigma Merck	D6944	00001124874	Rabbit	1:500
β actin	Sigma Merck	A5441	0000120483	Mouse	1:3000
CYP2E1	Invitrogen	PA535351	XE3583742	Rabbit	1:500
CYP2D6	Invitrogen	PA535148	XE3583741A	Rabbit	1:500
CYP1A1	Invitrogen	PA55213	XE3583745	Rabbit	1:500
CYP3A4	Invitrogen	PA514896	XE3583744	Rabbit	1:500
Secondary antibody Anti-Rabbit IgG–Peroxidase	Sigma Merck	A0545	L006328	Goat	1:2000
Secondary antibody Anti-Mouse IgG (H + L)-Hrp Conjugate	BioRad	1706516	L005680	Goat	1:3000

Info S1. Quantitative real-time PCR details

RNA extraction with with TRIzol™ reagent

Cells were scraped with 400 µl of TRIzol and after an overnight incubation at -80° C, cell lysate was centrifuged at 12,000 g (10 min, 4 °C), then the supernatant was transferred to a new sterile tube and mixed with 100 µl of chloroform and incubated on ice. After 30 minutes, the samples were centrifuged at 12,000 g (15 min, 4 °C). The top layer containing the RNA was transferred to a new sterile tube and mixed with 250 µl of isopropanol. After 30 minutes of incubation on ice, the samples were centrifuged for 8,000 g (15 min, 4 °C) and the pellet resuspended in 250 µl of 75% ethanol which was then centrifuged for at 6,000 g (5 min, 4 °C). The pellet containing the RNA was left air drying and then resuspended in 15-25 µl of nuclease-free water. Samples optic density (OD) at 260 and 280 nm was recorded to calculate the RNA concentration and only those with an OD260/280 ratio (degree of purity) between 1.8 and 2.1 were selected. RNA amount of PCR samples was determined with the NanoDrop ND-1000 UV-Vis Spectrophotometer by (Thermo Fisher Scientific Waltham, Massachusetts, USA), using the software ND-1000 V3.3.0 (Thermo Fisher Scientific Waltham, Massachusetts, USA).

RNA reverse transcription protocol

1 µg of total RNA from each sample was diluted in 10 µl of RNAse-free water. This RNA solution was mixed with 10 µl of master mix, transferred to a 200 µl tube and placed in a 9800 Fast Thermal Cycler (Applied Biosystems). Conditions of the reverse transcription is reported below.

	**Step 1**	**Step 2**	**Step 3**	**Step 4**
**Temperature (°C)**	25	37	85	4
**Time (min)**	10	120	5	∞

Thermal cycler conditions used for reverse transcription reactions. Cycles of time and temperature were set for a reaction volume of 20 μl.

RNA samples were transcribed to cDNA in a 9800 Fast Thermal Cycler from Applied Biosystems (Thermo FisherScientific Waltham, Massachusetts, USA). PCR was carried out in a StepOne™ Real-Time PCR System (Thermo FisherScientific Waltham, Massachusetts, USA) and data was acquired with the StepOne 2.0 software (Thermo FisherScientific Waltham, Massachusetts, USA).

The resulting cDNA was used for a PCR amplification according to manufacturer indications with slight changes. Briefly, 2 μl of cDNA samples were loaded in duplicate in the multi strip with 8 μl of mix solution and then placed in a StepOne™ Real-Time PCR System and analysed. The thermal conditions started with an initial holding stage of 20 seconds at 95 °C, followed by a two-step cycling stage, repeated for 45 cycles, 1 second denaturation at 95 °C and a subsequent annealing/extension step of 20 seconds at 60 °C.

Info S2. Probe sequence for each CYP isoform used for qRT-PCR assay

Gene	Probe sequence (5′-3′)
CYP1A1 "reverse"	TCTTGGATCTTTCTCTGTACC
CYP1A1 "forward"	CATTAACATCGTCTTGGACC
CYP2E1 "reverse"	TTCATTCAGGAAGTGTTCTG
CYP2E1 "forward"	GACACCATTTTCAGAGGATAC
CYP2D6 "reverse"	TTTGGAACTACCACATTGC
CYP2D6 "forward"	CCTATGAGCTTTGTGCTG
CYP2B6 "reverse"	TTTCCATTGGCAAAGATCAC
CYP2B6 "forward"	AGGTTCCGAGAGAAATATGG
CYP3A4 "reverse"	ACATAATGAAGGGGAGAGTG
CYP3A4 "forward"	TAAAGCTCTGTCTGATCTGG
RPS 18 "reverse"	TATTTCTTCTTGGACACACC
RPS 18 "forward"	CAGAAGGATGTAAAGGATGG

Probes were selected from the manufactured inventory of by Sigma Merck (Darmstadt, Germany).
